# Psoas abscess secondary to retroperitoneal distant metastases from squamous cell carcinoma of the cervix with thrombosis of the inferior vena cava and duodenal infiltration treated by Whipple procedure: A case report and review of the literature

**DOI:** 10.1186/s12893-016-0169-7

**Published:** 2016-08-11

**Authors:** Matthias Mehdorn, Tim-Ole Petersen, Michael Bartels, Boris Jansen-Winkeln, Woubet Tefera Kassahun

**Affiliations:** 1 Department of Surgery II, Faculty of Medicine, Clinic for Visceral, Transplantation, Thoracic and Vascular Surgery, OKL, University of Leipzig, Liebig Strasse 20, Leipzig, 04103 Germany; 2 Department of Radiology, University of Leipzig, Liebig Strasse 20, Leipzig, 04103 Germany

**Keywords:** Malignant psoas abscess, Squamous cell carcinoma of the cervix, Whipple procedure

## Abstract

**Background:**

Psoas abscess is a rare clinical disease of various origins. Most common causes include hematogenous spread of bacteria from a different primary source, spondylodiscitis or perforated intestinal organs. But rarely some abscesses are related to malignant metastatic disease.

**Case presentation:**

In this case report we present the case of a patient with known squamous cell carcinoma of the cervix treated with radio-chemotherapy three years prior. She now presented with a psoas abscess and subsequent complete inferior vena cava thrombosis, as well as duodenal and vertebral infiltration. The abscess was drained over a prolonged period of time and later was found to be a complication caused by metastases of the cervical carcinoma. Due to the massive extent of the metastases a Whipple procedure was performed to successfully control the local progress of the metastasis.

**Conclusion:**

As psoas abscess is an unspecific disease which presents with non-specific symptoms adequate therapy may be delayed due to lack of early diagnostic results. This case report highlights the difficulties of managing a malignant abscess and demonstrates some diagnostic pitfalls that might be encountered. It stresses the necessity of adequate diagnostics to initiate successful therapy. Reports on psoas abscesses that are related to cervix carcinoma are scarce, probably due to the rarity of this event, and are limited to very few case reports. We are the first to report a case in which an extensive and complex abdominal procedure was needed for local control to improve quality of life.

## Background

Psoas Abscess is a rare disease with a multitude of etiologies. It can be divided into primary and secondary abscesses each with different etiology and predominant age prevalences. Primary abscesses are mainly due to a hematogenous spreading of bacteria. In western countries primary psoas abscess mostly occurs in immunocompromised patients such as diabetics, alcoholics and HIV positive patients ^1,2^. The most frequent microbial cause of psoas abscess is Staphylococcus aureus [1]. Secondary psoas abscess originates from perforated intestinal organs such as perforation in diverticulitis [2], perforated appendicitis [3] or perforation secondary to colon cancer [2, 4]. Other causes include direct spread of inflammation seen in pyelonephritis or spondylodiscitis [5] with the latter being the most frequent cause of abscedation. Consequently bacteria from the intestinal gram-negative flora such as escherichia coli, enterobacter, clostridia and bacteroides can be found [6]. In the case of spinal infection, staphylococcus aureus and mycobacterium tuberculosis are the pathogens mostly found in the abscess [5].

Patients mostly present with non-specific symptoms such as back or hip pain, fever, general malaise or weight loss [7]. Difficulty of making the correct diagnosis can be attributed to its non-specific presentation as well as its rarity. As a result appropriate diagnostics are often delayed leading to inadequate treatment [8]. Treatment of the primary cause remains the only curative treatment.

Only very few cases of malignant psoas abscess secondary to metastatic squamous cell carcinoma can be found in literature which are presented in Table 1. All those patients presented with non-specific symptoms such as thigh pain or fever. In most of the cases several diagnostic steps were necessary to make the correct diagnosis and initiate the appropriate therapy. We report a case of malignant psoas abscess secondary to metastatic cervical cancer which required an extensive abdominal procedure.

**Table 1:** Summary of literature showing cases of psoas abscess in metastatic squamous cell carcinoma of cervix

Authors	Age	FIGO Stage of SCC	Symptoms at presentation at hospital	Primary diagnosis	Diagnostic tools in order of application	Initial Treatment of cervical cancer	Treatment for metastases/abscess	Microbiology of psoas abscess	Complication of metastases
Singh et al. [11]	24	IIIB	Vaginal discharge, abdominal pain	Tuboovarian abscess, pelvic inflammatory disease and AIDS	CT, FNAC	Patient refused radiation	No specific	Sterile	None
George, Lai [14]	60	IIB	Left flank mass, backpain	Metastatic renal cell carcinoma	Lumbar X-ray, ultrasound, MRI, CT-guided FNAC	radiotherapy	Surgical drainage	Sterile	Left kidney loss of function, vertebral infiltration L1-L3
Bar-Dayan et al. [20]	50	NA	NA	NA	CT-guided FNAC	chemotherapy	Intralesional chemotherapy	NA	Iliac bone distruction
Lüring et al. [18]	36	NA	Back pain	Pregnancy associated back pain	Ultrasound, MRI, sonogrpahy guided drainage and cytology	chemotherapy	Sonography guided drainage	Sterile	Iliac bone and nerve infiltration
Kalra et al. [12]	60	IIB	Back pain	Tuberculoid psoas absces	CT, MRI, CT-guided biopsy	Radiochemotherapy	Radiotherapy after histological finding	Sterile	Vertebral infiltration
Basu, Mahajan [19]	52	IIIB	Thigh pain	NA	FDG-PET/CT, MRI, FNAC	Radiochemotherapy	Radiochemotherapy	NA	None
Askin et al. [8]	54	NA	Hip pain, fever	Psoas abscess	CT	Radiochemotherapy	Piperacillin/Metronidazole, resection of abscess, ureter and sigmoid colon	Bloodculture sterile	Ureter and sigmoid colon infiltration

*CT* computed tomography, *FIGO* International Federation of Gynecology and Obstetrics, *FNAC* fine-needle aspiration cytology, *MRI* magnetic resonance imaging, *NA* not available, *SCC* squamous cell carcinoma

## Case presentation

A 51 year-old female patient was admitted to our emergency department due to fever and general malaise.

Three years earlier, the patient had been treated for locally advanced squamous cell carcinoma of the cervix. The initial stage of the tumor was reported to be TNM; T3b-T4a, N1, M0; International federation of gynecology and obstetrics; FIGO stage IIIb based on pelvic MRI and whole body PET-CT scan. There was an involvement of the parametria, upper third of the vagina, external compression of the right ureter causing hydronephrosis (biopsy of the pelvic sidewall on the right side revealed no cancer) and three suspicious iliac lymph nodes. She was treated with a curative intent using concurrent chemo-radiotherapy. For external beam radiotherapy, whole pelvic irradiation of 50.4 Gy was delivered in 25 fractions over 5 weeks as intensity modulated radiotherapy concurrently with chemotherapy (Vinorelbin) followed by boost irradiation to the tumor region of 9 Gy in 5 fractions. Weekly high-dose rate intracavitary brachytherapy delivered 20 Gy in 4 fractions (over a duration of 5-weeks). Follow up indicated no evidence of local recurrence or metastatic disease, with the exception of an enlarged paracaval lymph node.

**Fig. 1:**
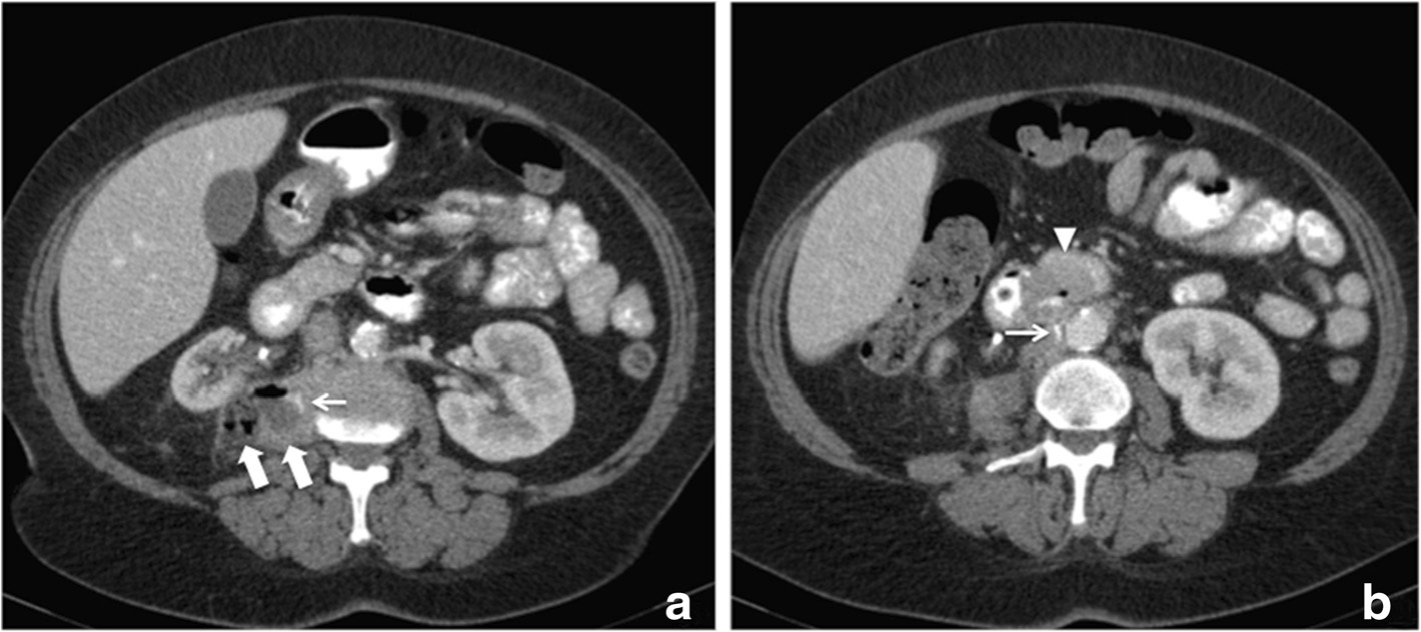
Abdominal CT scan at admission. Axial slices of a contrast enhanced computed tomography showing a 29 x 41 mm abscess (thick white arrow) in the right psoas muscle (**a**). Positive oral contrast media marking the fistula (small white arrow) from the thickened wall of the duodenum (white arrowhead) to the abscess (**b**)

Initial laboratory investigation demonstrated an elevated leukocyte count and C-reactive protein. A CT-scan showed an abscess formation in the right psoas muscle and retroperitoneum with connection to the right kidney capsule and a fistula with intestinal lumen (Fig. 1). A CT guided interventional drain was placed. Microbiological culture grew Citrobacter freundii, Enterococcus faecalis and Prevotella buccae. All of those belong to the bacterial flora of the intestine suggestive of intestinal perforation. An antibiotic regime with sultamicilline/clavulanic acid was started in accordance with the findings of microbiology. We irrigated the abscess continuously. This resulted in a significant decrease of the initially elevated inflammatory parameters. Additionally an endoscopic examination was performed to further investigate the suspicion of a duodenal perforation. This confirmed a duodenal ulcer with covered perforation at the ventral side of the second part of the duodenum. Biopsies showed cell formations in accordance with an edge of a perforated ulcer but no malignancy. Closure with an over the scope clipping (OTSC) and high-dose proton pump inhibitor therapy resulted in local control and significant clinical improvement. Surgical intervention was not intended at that point because the abscess decreased in size. After further improvement of the clinical condition of the patient, she was discharged to follow-up with the drains in place.

**Fig. 2:**
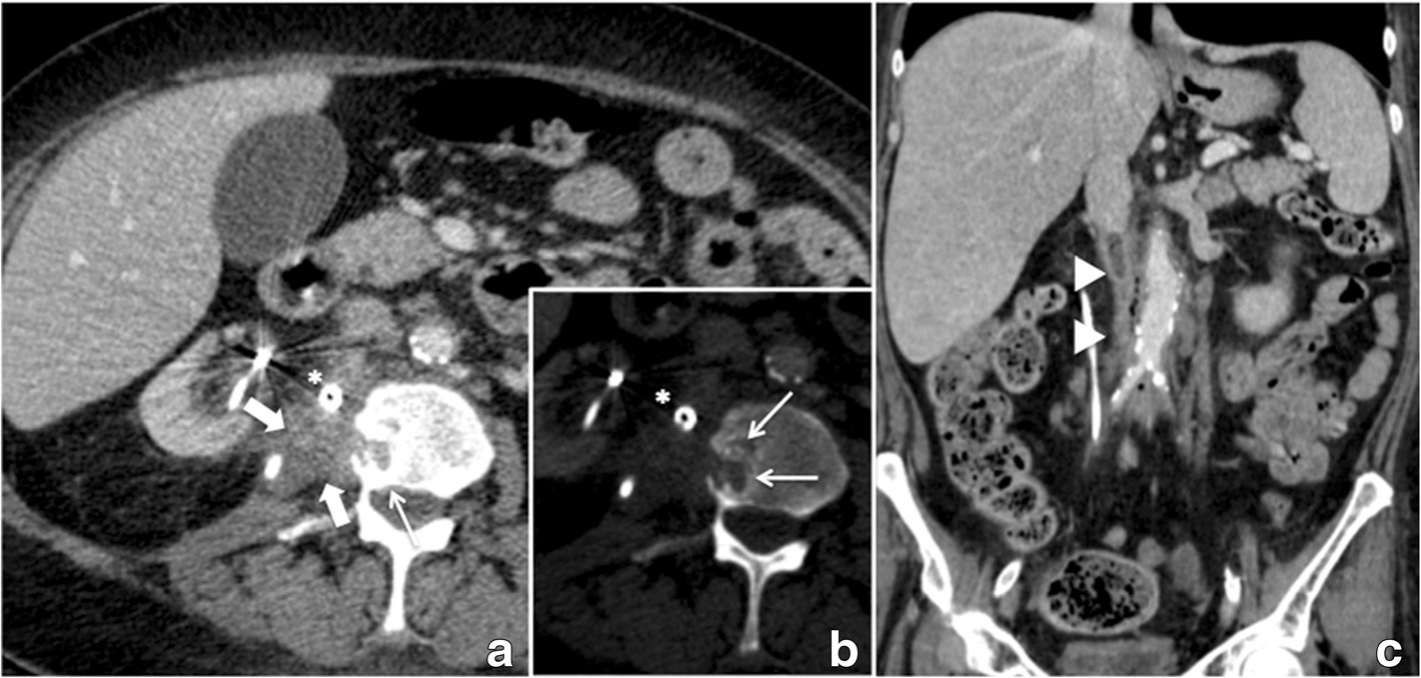
Initial follow-up CT scan 2 months after treatment with percutaneous drainage showing a marked decrease in size of the abscess (thick white arrow; **a**), however, Osteolytic bone lesion in adjacent second lumbar vertebra (small white arrows **a** and **b**), a streaky densification of the pericaval connective tissue and a nearly complete thrombotic occlusion of the infrarenal inferior vena cava (white arrowhead **c**)), which was probably infected (small gas bubbles). Infection related air bubbles in the retroperitoneal space can also be seen now along the aorta to below the aortic bifurcation

**Fig. 3:**
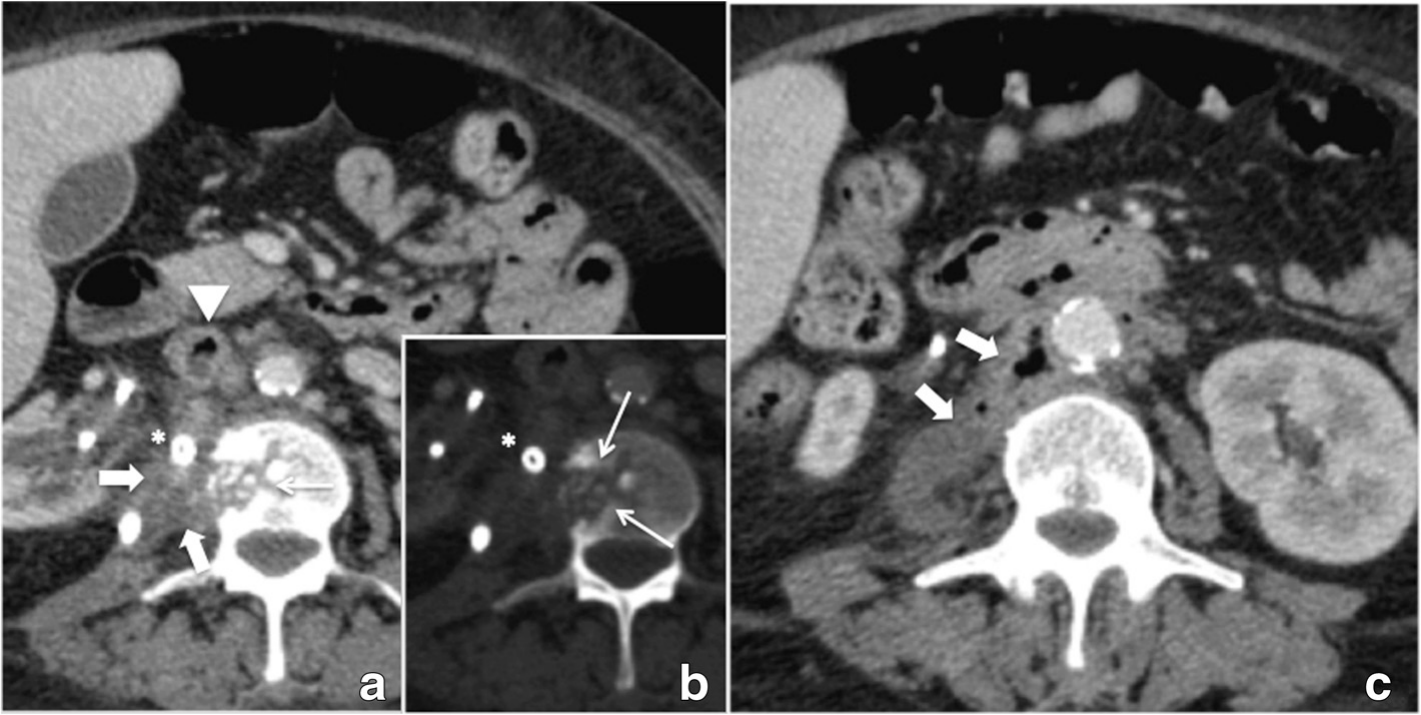
consecutive follow-up CT scan showing constant size of the abscess (thick white arrow) (**a**, **c**), progressive osteolytic lesion (small white arrow) (**b**), and infectious thrombus in the inferior vena cava (white arrowhead)

Two months after discharge she presented with a transient ischemic attack (TIA) to our neurology department caused by a high grade stenosis of the left internal carotid artery. She was treated with carotid endarterectomy in our vascular surgery department and commenced on aspirin. Because of continuous and partially bloody secretion from the drain we performed a follow up gastroscopy and CT-Scan which showed no intraluminal duodenal changes and a continuous decrease in the size of the ulcer. It did however note regional progress of the known psoas abscess and partial destruction of the second lumbar vertebral body (Fig. 2) which was interpreted as osteitis. Furthermore, imaging revealed an involvement of the inferior vena cava and aorta in the abscess with discrete retroperitoneal air attributed to inflammation. Antibiotic therapy was administered with piperacilline/tazobactame in accordance with microbiological findings from the drain of Klebsiella pneumoniae, Escherichia coli and still Enterococcus facalis and Citrobacter freundii. The patient was discharged again but returned to our emergency department two weeks later complaining of back pain radiating to her right thigh. Blood tests demonstrated newly elevated inflammatory parameters and anemia. This time a CT-Scan showed progression of the vertebral body destruction and a complete thrombosis of the inferior vena cava with an inflammatory reaction of the vessel (Fig. 3). In view of the high risk of life threatening hemorrhage from possible perforation of the inferior vena cava we decided on an explorative laparotomy. This revealed a massive tumorous infiltration of the duodenum. The tumor was closely attached to the surrounding organs; therefore the only reasonable treatment option was a pylorus preserving partial duodenopancreatectomy (Whipple procedure). Cellular pathology reported a metastatic cervical squamous cell carcinoma. After an uneventful postoperative course the patient was discharged two weeks post-surgery. Following surgery, the case of the patient was discussed in our interdisciplinary tumor board and palliative chemotherapy was recommended. However, due to multiple comorbidities and the patient’s suboptimal performance status, chemotherapy was not performed. During the last 12 months of follow-up period, the patient showed no clear evidence of an extensive local recurrence or distant metastatic disease. However, the patient is still suffering from chronic back pain related to spondylodiscitis at L2/L3. After orthopedic evaluation surgical therapy was recommended. However, she refused further surgery and opted for outpatient antibiotic treatment.

## Conclusion

Squamous cell carcinoma of the cervix mostly infiltrates pelvic organs directly but it also frequently metastasizes to the paracaval retroperitoneum [9]. Similar to our case about 31 % of stage III cervical carcinomas show local tumor recurrence after radiochemotherapy [10]. Occurrence of retroperitoneal metastases has been considered an aggressive form of cervix carcinoma predominantly seen in HIV-positive women [11]. Retroperitoneal metastases may infiltrate intestine or vertebrae [12–14]. This may result in extensive tumor necrosis and abscess formation as in the presented case and other case reports (Table 1).

The diagnostic approach to psoas abscess is complicated because of its non-specific symptoms which may be misleading. Therefore diagnostic steps vary widely as underlined by the cases in Table 1. Ultrasound and X-ray may help raise suspicion of psoas abscess but the final diagnosis is mostly established by CT-scan which is considered the gold-standard [2]. Previous studies have shown that muscle metastases might be interpreted as an abscess as around 27.7 % tend to appear like an abscess on CT-images [15]. None of the cases presented had metastases of cervical carcinoma as primary diagnosis. Only histological sampling may confirm the primary cause of the abscess. In all of the reported cases microbiological culture of the aspirate was negative (Table 1). Nevertheless some patients were initially treated with antibiotics until a final diagnosis was established as they presented with elevated inflammatory parameters. In contrast to the cases in Table 1 we obtained a positive microbiological culture which was probably due to duodenal perforation.

In the context of infectious psoas abscess the therapeutic standard is image-guided drainage accompanied by sufficiently long-term antibiotic therapy [16]. In literature controversy exists whether percutaneous drainage is superior and more successful compared to open surgical drainage. With modern interventional radiology a CT guided percutaneous drainage is a feasible and safe intervention to perform [1, 2, 16]. It has been suggested, however, that patients with percutaneous drainage more often show recurrence if the abscess is accompanied by a higher degree of inflammation presenting on the CT-scan by inclusion of air [6, 17]. Image-guided drainage and open drainage was performed in some of the cases in Table 1 [14, 18]. Our patient was also treated with a CT-guided drain which remained in place for almost four months. Although the abscess showed a decrease in size it never resolved fully which was attributed to the malignant origin of the disease. From the other cases it is evident that only a correct histological classification of the abscess could lead to an appropriate treatment of the primary cause which is concurrent chemoradiotherapy [12, 19, 20].

The surgical exploration in our patient was undertaken because of the suspected risk of major bleeding given the sudden onset of anemia within two weeks. This might have been caused by bleeding from the gastroscopically shown duodenal ulcer/perforation. Kanthan et al. have reviewed gastro-intestinal metastases of squamous cell carcinoma of the cervix that presented with gastro-intestinal bleeding [13]. Only three of those bleeds were localized in the duodenum. As the second possible cause of bleeding we suspected hemorrhage from the inferior vena cava which was thrombosed and involved in the inflammatory process as shown by CT-imaging. The nearly complete thrombotic occlusion of the inferior vena cava is may be related to a compression of the vein by surrounding inflamed connective tissue that led to an abnormal blood flow. Data in the literature supports this assumption [21, 22]. Proposed therapeutic regimens for such an inflammatory thrombus are anticoagulation and drainage of the adjacent abscess [22] or percutaneous thrombectomy with anticoagulation and antibiotic therapy [21]. However, in the case of inflammatory thrombosis secondary to metastatic disease this therapeutic approach is ineffective as demonstrated in the presented case.

Combining the information provided in literature and analyzing the course of our case retrospectively, an early operation would have been the most suitable approach to the psoas lesion. The treatment in our case was influenced by some misleading results such as the radiological interpretation of the lesion as being an abscess, the positive bacterial culture of the abscess and endoscopy showing a perforated duodenal ulcer without histological confirmation of malignancy. This case reiterates the complexity of psoas abscess regarding diagnosis and therapy. In order to be able to treat a malignant abscess successfully it is of utmost importance to obtain a sufficient histological specimen. This may allow applying a suitable therapeutic strategy without delay.
